# Composite-Free Vascularized Fibular Epiphyseal Flap and Proximal Humeral Allograft for Proximal Humerus Reconstruction in a Pediatric Patient

**DOI:** 10.5435/JAAOSGlobal-D-21-00009

**Published:** 2021-07-07

**Authors:** Matthew T. Houdek, Elizabeth P. Wellings, Hiba Saifuddin, Steven L. Moran

**Affiliations:** From the Department of Orthopedic Surgery, Mayo Clinic, Rochester, MN (Dr. Houdek and Dr. Wellings); Mayo Clinic School of Medicine, Rochester, MN (Saifuddin); and the Division of Plastic and Reconstructive Surgery, Mayo Clinic (Dr. Moran).

## Abstract

Tumors involving the epiphysis in children present a reconstructive challenge. A free vascularized fibula epiphyseal transfer offers a means for biological reconstruction and longitudinal growth; however, it is often complicated by graft fracture and limited shoulder motion. Here, we present a case of a composite structural allograft with free vascularized fibula epiphyseal transfer for proximal humeral reconstruction. At 27-month follow-up, there was longitudinal growth, hypertrophy of the epiphysis, shoulder function which allowed activities of daily living, and no graft fracture.

Reconstruction of bone defects after tumor resection in the pediatric population can be challenging. In some cases, the physis must be sacrificed, making reconstruction complex as the patient is at risk for developing a limb length discrepancy.^1^ The proximal humerus is responsible for 80% of humeral growth.^2-4^ Free vascularized epiphyseal transfer (FVET) has been used to reconstruct the humerus in pediatric patients; however, it is complicated by high fracture rates.^1,5-7^ Combined structural allografts and free fibula flaps can protect the fibula from facture and restore bone stock.^8,9,10,11,12,13,14^ Here, we present a case of a pediatric proximal humeral reconstruction using the combination of a FVET and structural allograft with rotator cuff tendon after en bloc resection. Informed verbal consent was obtained from this patient's family before completion of this case report.

## Case Report

**Figure 1:**
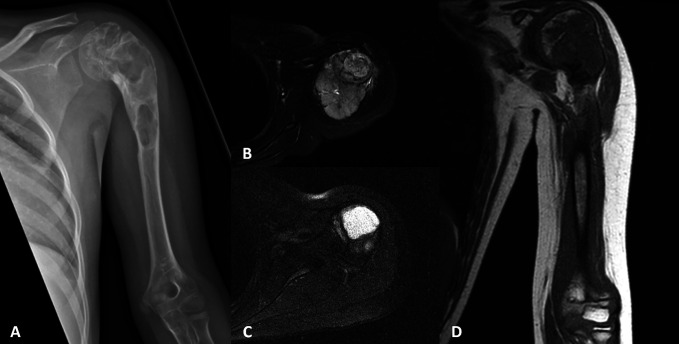
Preoperative radiographs (**A**) showing a proximal humeral Ewing sarcoma. Before the induction of chemotherapy, the patient had a large soft-tissue mass (**B**) on T2-weighted MRI, which resolved before surgery (**C**). After chemotherapy on coronal T1-weighted MRI (**D**), there is marrow replacement in the physis after chemotherapy.

A 9-year-old girl was referred to our center with an Ewing sarcoma of the proximal humerus. Initially, she underwent curettage and cementation at an outside center for a pathological fracture of what was thought to be a benign cyst. The tumor progressed, and a biopsy showed Ewing sarcoma, which was confirmed by FISH for EWSR1 rearrangement, involving the proximal humeral metaphysis and epiphysis (Figure 1). She was treated with neoadjuvant chemotherapy consisting of vincristine, cyclophosphamide, doxorubicin, ifosfamide and etoposide, and proton radiation therapy because of the soft-tissue contamination. A FVET was planned supplemented with a fresh frozen allograft and rotator cuff.

**Figure 2:**
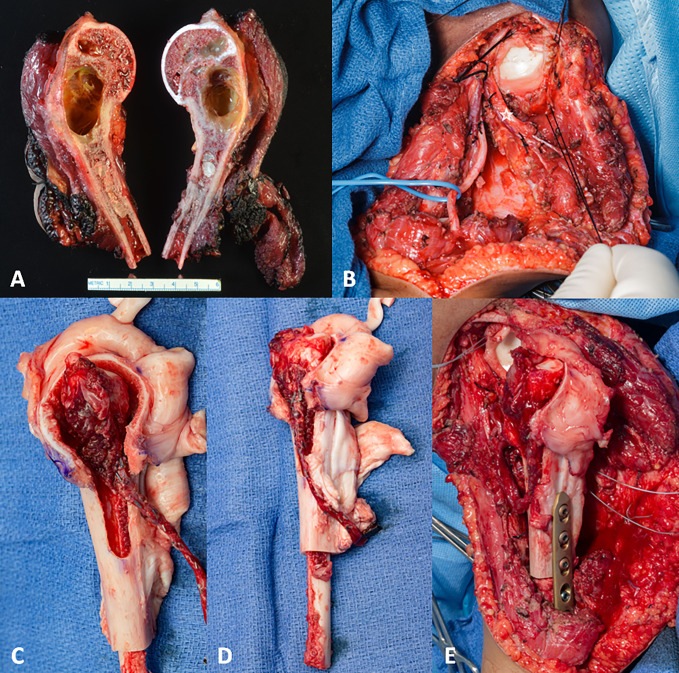
Photograph of the specimen that was removed en bloc showing involvement of the physis (**A**) with a portion of the anterior deltoid and triceps because of the previous inadvertent procedure and residual soft-tissue extension. The radial nerve (loop) and axillary nerve branches (star) were preserved going to the posterior deltoid (**B**). The fresh frozen allograft is prepared by removing the articular surface of the humeral head and clearing the cancellous bone from the metaphysis, with a window created to allow for the vascular pedicle (**C**). This allows room from the proximal fibula epiphysis to be harvested with a cuff of muscle to protect the recurrent epiphyseal vessels and the FVET is intussuscepted into the allograft. The distal portion of FVET was then intussuscepted host humerus, allowing for the articular surface of the fibula to face the glenoid (**D**). The anastomosis was completed showing bleeding from the physis and the distal fibula was intussuscepted into the humerus and the allograft fixed with a compression plate (**E**). FVET = free vascularized epiphyseal transfer

The proximal humerus was approached using an extended deltopectoral approach, and an intra-articular resection of the proximal humerus was performed (Figure 2). The tendons of the rotator cuff cut at the myotendinous junction and tagged for repair. Owing to the previous nononcologic surgery, the anterior half of the deltoid and accompanying axillary nerve were resected. A FVET was harvested with the anterior tibial vessels.^15^ The articular surface of a cadaveric proximal humerus was removed with a saw, and the cancellous bone of the metaphsysis was removed with a burr to the cortical bone to allow for fitting of the fibular head, whereas the diaphysis was reamed to allow for the FVET to be intussuscepted within the humerus allograft with the articular surface of the fibula facing the glenoid at the level of the tuberosity. A window was created for the vascular pedicle on the medial aspect of the allograft. The anterior tibial artery was anastomosed to a side branch of the profunda brachii artery and vein. The distal end of the fibula was intussuscepted into the remaining host distal humerus, and the allograft was fixed with a compression plate. The remaining host rotator cuff was attached to the allograft cuff via heavy suture.

After fibula harvest, the lateral collateral ligament of the knee was reconstructed via fixation of the biceps tendon to the proximal tibia with heavy suture. A single branch of the deep peroneal nerve to the proximal anterior tibialis muscle belly was divided with subsequent primary repair to allow for harvest of the proximal head of the fibula.

Margins were negative, and a greater than 95% tumor necrosis was noted. Postoperatively, they had an expected anterior tibial muscle palsy, which was treated with a temporary standard ankle-foot orthosis. She was placed in a shoulder immobilizer for 3 months, preventing active shoulder motion; however, she could begin passive and active elbow, hand, and wrist motion after surgery. After the immobilization, she could use the arm as tolerated. Chemotherapy was resumed, and the allograft/host junction was found to be healed at 9 months postoperative (4 months after the completion of chemotherapy).

**Figure 3:**
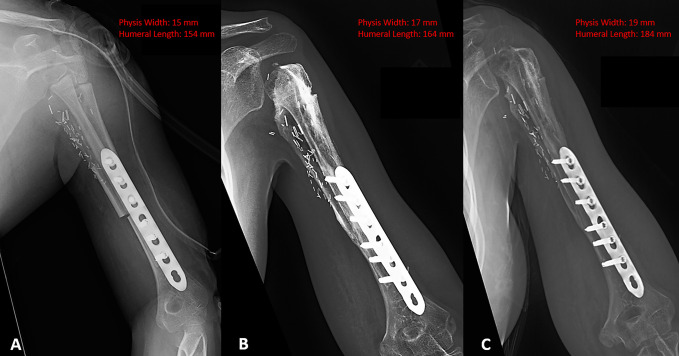
Immediate postoperative magnification correction radiographs showing a physis width of 15 mm and a humeral length of 154 mm (**A**) as measured from the top of physis to the distal olecranon fossa. At 15-month postoperative, the allograft/host junction had healed and the physis became spherical, with a width of 17 mm and humeral length of 164 mm (**B**), and at 27 months postoperative, the width was 19 mm with a humeral length of 184 mm (**C**).

**Figure 4:**
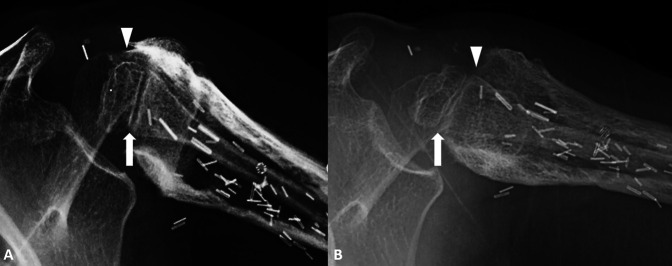
Axillary view radiographs show an open physis (arrow) with the tip of the epiphysis below the proximal portion of the allograft (arrowhead) at the 15-month follow-up period (**A**). At 27 months postoperative, the physis remained open (arrow) with the tip of the epiphysis now above the proximal portion of the allograft (arrowhead), highlighting the growth of the proximal humerus (**B**).

At the most recent follow-up (27 months postresection), there was no evidence of tumor recurrence based on whole body PET-CT. She had no pain and had resolution of her peroneal palsy, with 60° of forward elevation and abduction of 50° but limited external rotation in the left shoulder—with full motion of her elbow and use of her hand. Her Musculoskeletal Tumor Society^16^ rating was 80%, and her American Shoulder and Elbow Surgeons Shoulder Score^17^ was 75%. Radiographs showed healing of the allograft junction; however, resorption of the allograft was present. The FVET showed evidence hypertrophy and remodeling of the epiphysis and longitudinal growth of approximately 3 cm (Figure 3) in addition to an open physis (Figure 4).

## Discussion

Physeal transfers via free vascularized fibula flaps have been well reported, resulting in the successful creation of a neoglenofibular joint. However, these constructs are known to be complicated by early graft fracture.^1,6,18,19^ The addition of allograft to a vascularized fibular physis has not been reported on in the upper extremity. It was previously believed that the proximal humerus did not need additional structural support because the vascularized fibula has been shown to hypertrophy and approach the diameter of the humerus; however, graft fracture is common.^1,6,18,19^ In this case report, the additional structural support of an allograft helped prevent graft fracture within the first-year postoperatively while maintaining an open physis and allow for epiphyseal hypertrophy and growth. In addition, it allowed for repair of the rotator cuff to allow for some forward elevation of the arm.

Free vascularized fibula flaps are widely used for many reconstructive scenarios, including physeal transfer first described by Innocenti et al.^20^ They are beneficial compared with other vascularized grafts because of the presence of an articular surface for creation of a neoarticular joint and a tubular structure for reconstruction of long bone defects.^6,18,21^ In the proximal humerus, survival of the physis and creation of a neoglenofibular joint have been successful, but a high rate of graft fracture is noted, usually within the first year before enough graft hypertrophy has occurred because of the diameter mismatch between the fibula and humerus.^1,5-7^ One study looked at 11 proximal humerus physeal transfers and reported a 66% graft fracture rate within the first year.^1^

The Capanna technique is used for reconstruction of intercalary defects of lower extremity long bones to support weight-bearing. The concept is based on the combination of a structural allograft used for initial support with an autograft capable of internal repair without stress shielding.^9^ Because the fibula gradually hypertrophies over 1 to 3 years, it is able to compensate for the allograft resorption,^9^ as is being observed in our patient. In pediatric patients, this technique is a reliable method with an acceptable complication rate for intercalary resections.^10,14^ One known case report performed a FVET and allograft technique in the proximal femur.^11^ At the 9 months follow-up, the allograft had united; however, it did not seem the physis remained viable. It should be noted that the fibula was harvested on the peroneal vessel, which does not reliably vascularize the physis.^15^

Physeal viability and long bone growth can be confirmed by measuring from the tip of the epiphysis to the distal aspect of the plate or screws. Our graft demonstrated approximately 3 cm of longitudinal growth in the first 27 months after transfer. This is consistent with previous studies showing an annual growth rate of 0.7 to 1.35 cm per year in 24 patients including proximal humerus and distal radius physeal transfers.^6^ In the series, five cases had premature physeal closure and five had graft fracture.^6^

Shoulder function after FVET has been underreported. In the series by Stevenson et al,^1^ the authors used the Musculoskeletal Tumor Society and Toronto Extremity Salvage Score, which does not focus on activities of the shoulder, rather on the upper extremity in general.^16,22^ In this study, our patient had resection of the anterior portion of the deltoid and axillary nerve, which is important for forward elevation of the shoulder.^23^ Since the allograft was supplemented with rotator cuff, it allowed for a primary repair of host rotator cuff to the allograft, allowing for some forward elevation and a functional shoulder based on the American Shoulder and Elbow Surgeons Score. To improve forward elevation, newer options for tendon transfers are available, namely upper pectoralis major transfer;^24^ however, their use in the oncologic setting is limited.

A transient peroneal palsy and foot drop is to be expected as branches of the deep peroneal nerve may need to be divided and repaired to disarticulate the fibular head during harvesting.^15,21^ Rarely is the foot drop permanent (2.6%).^21^ At the follow-up, the patient's range of motion was limited but as expected. A series of 11 patients after proximal humerus physeal transfer with only vascularized fibular grafts found a mean abduction, forward flexion, and external rotation of 57°, 63° and 19° at the 5-year follow-up.^1^

In this study, a composite allograft with a FVET was successfully performed providing limb salvage, physis survival and acceptable functional outcomes at 27-months post-resection. Further follow-up is needed to determine long-term efficacy of this procedure, as longitudinal growth may not be the only answer for these patients. Long-term changes in glenoid morphology need to be evaluated to determine if this is the optimal treatment of these patients. It is possible that with further follow-up, it may be shown that other forms of reconstruction maybe more efficacious.
